# Research on the Impact of Members’ Social Capital within Agricultural Cooperatives on Their Adoption of IPM in China

**DOI:** 10.3390/ijerph191811538

**Published:** 2022-09-13

**Authors:** Yuying Liu, Ziqi Liu, Jingzheng Liu, Ling Qiu, Yulin Wang, Xinhong Fu

**Affiliations:** College of Management, Sichuan Agricultural University, Chengdu 611130, China

**Keywords:** IPM adoption, agricultural cooperatives, mediating effect, green production technology, IV probit model

## Abstract

Agricultural cooperatives are effective facilitators of green production technology promotion. What is the role of social capital within agricultural cooperatives with the most competitive advantage in technology promotion? Using the survey data of 465 citrus-planting cooperative members in Sichuan Province, this study uses the IV-probit model and mediating effect model to analyze the impact role of social capital within agricultural cooperatives on its members’ adoption of integrated pest management (IPM) technology. The bootstrap method is also used to test the robustness of the parameter estimates. The results show that: (1) the social capital within agricultural cooperatives has a significant positive impact on IPM adoption; (2) cooperative members’ IPM cognition has a partial mediating effect on the impact of the social capital within agricultural cooperatives on its members’ adoption of IPM technology (more than 51.37%). Therefore, among all the optional IPM technology promotion measures of cooperatives, multi-dimensional accumulation of the social capital within agricultural cooperatives and promotion of IPM technology awareness level of members is a viable path.

## 1. Introduction

Adopting conventional chemical pesticides has long been regarded as an effective way to save labor and improve agricultural production efficiency [1]. However, long-term misuse of conventional chemical pesticides not only increases production costs but also endangers the soil and water environment and the quality and safety of agricultural products [2,3]. As a green production technology, integrated pest management (IPM) technology is considered an effective alternative to pesticides to achieve increased agricultural production and income with minimal environmental cost [3]. The farmer is the basic unit of agricultural production and operation, so their adoption of IPM technology is the key to the popularity of IPM technology. Due to the lack of awareness of green agriculture, the ability to adopt green technologies, and marketing channels for green agricultural products, it is difficult for smallholder farmers to adopt IPM technologies consciously.

As a cooperative economic organization voluntarily formed by farmers, agricultural cooperatives are believed to be effective in promoting farmers’ adoption of agricultural production technologies [4]. For example, Li et al. argued that agricultural cooperatives could motivate farmers to engage in safe production based on collective action theory [5]. Based on data from vegetable farmers in Shandong, China, Yang et al. confirmed that agricultural cooperatives could effectively reduce pesticide use [6]. Liu and Wu further analyzed that cooperatives could reduce pesticide use through production and marketing services [7]. In particular, based on data from Chinese apple farmers, Ma and Abdulai showed that membership in agricultural cooperatives could increase the likelihood of IPM technologies adoption [3]. However, few studies focus on the role of social capital within agricultural cooperatives in promoting the adoption of IPM technologies by farmers. As an agricultural cooperative embedded in a rural social network, social capital is its most fundamental asset [8] and the most competitive capital [9]. Scholars have found that the social capital within agricultural cooperatives can enhance cooperative members’ satisfaction with cooperative management [10] and the farmers’ possibility of participating in agricultural cooperatives [11]. Therefore, can the social capital within agricultural cooperatives influence the adoption of IPM techniques by its members or not? What is the mechanism by which this may occur?

The primary objective of this study is to analyze the effect of social capital within agricultural cooperatives on the adoption of IPM techniques by its members and how this occurs. This study contributes to the literature on the development of agricultural cooperatives and farmers’ production behaviors in three ways. First, although it has been confirmed that agricultural cooperatives can effectively influence pesticide use reduction and IPM technology adoption among its members, the impact of agricultural cooperatives is chiefly measured directly using the indicator of “whether or not to join a cooperative”. Not all agricultural cooperatives actively promote the adoption of green production technologies by their members, and not all members in the same agricultural cooperative adopt IPM technologies in the same way. Therefore, this study investigates the influence of social capital within agricultural cooperatives on the farmers’ adoption of IPM technologies. Second, although previous studies have pointed out that the social capital of agricultural cooperatives has an essential influence on its members’ production and management behavior, few studies analyze the paths of the social capital within agricultural cooperatives on IPM technologies adoption by its members. This study analyzes the impact mechanism of social capital within agricultural cooperatives on its members’ IPM technology adoption behavior from a cognitive perspective. Third, this study uses an IV-probit model to examine the effect of the social capital within agricultural cooperatives on the adoption of IPM technology by controlling for the endogeneity problem. Furthermore, using a mediating effect model explores the mediating role of technology perception to provide theoretical support and empirical evidence to enhance the possibility of farmers’ adoption of IPM technology from the perspective of accumulating social capital within agricultural cooperatives. 

The remainder of the study design is as follows. Section 2 presents our theoretical analysis to identify how the social capital within agricultural cooperatives affects agricultural cooperatives’ members’ IPM adoption behavior. Section 3 introduces the empirical model with variables selected, then describes the data used. The empirical results and discussion reports are in Section 4. Conclusions and policy implications are presented in the final section.

## 2. Theoretical Analysis and Research Hypotheses

### 2.1. The Effect of Social Capital within Agricultural Cooperatives on IPM Technology Adoption by Their Members 

According to social capital theory, the social network can help individuals build trust, cooperate, and obtain resources such as information, capital, and technology in the social network [12]. Adler divided social capital into internal and external social capital, in which internal social capital refers to the institutional norms, relationship networks, and trust among members within the organization; external social capital is mainly used to measure the relationship between the organization and external stakeholders [13]. The internal social capital of agricultural cooperatives mainly refers to the trust, norms, and relationship network among cooperatives’ members. According to Adler and Kwon’s opportunity-motivation-ability framework [13], an actor’s network of social ties creates opportunities for social capital transactions, and trust and norms are two key motivational sources of social capital. Social networks allow actors to leverage their contacts’ resources and create the opportunity to act together. Norms resolve collective action problems, bind communities, and transform individuals from self-seeking and egocentric agents with little sense of obligation to others into members of a community with shared interests, a common identity, and a commitment to the common good. Trust ensures social networks are periodically renewed and reconfirmed and keeps their efficacy. Thus, this study argues that improving the social capital within agricultural cooperatives can enable their members to be in a closed social network. Moreover, members can participate in the dissemination of information, share marketing and technology information, and obtain more social resources to master new technologies in this social network. Thus, agricultural cooperatives’ members are more likely to adopt a new production technology known by the agricultural cooperative.

Referring to the existing studies [10,12,14], the internal social capital of agricultural cooperatives mentioned in this study mainly refers to the social capital within agricultural cooperatives primarily owned by agricultural cooperatives’ members, which can be divided into three aspects: social network, internal trust, and internal norms. Then, the influence of the social capital within agricultural cooperatives on the adoption of IPM technology by members can be analyzed from the following three aspects.

The social network within agricultural cooperatives is an essential platform for interaction and learning among agricultural cooperatives’ members. When farmers choose to join agricultural cooperatives, their social networks expand. Expanding their social network will significantly help farmers obtain labor, information, and financial support in the production process, which could promote technical exchange among farmers and significantly promote farmers’ adoption of green production technologies [15]. In terms of internal trust, the higher the level of farmers’ trust in their relatives, neighbors, village cadres, and institutions, the more conducive it is to establishing information-sharing mechanisms and consensus among members [14]. Such an information-sharing mechanism is conducive to effectively disseminating value information, marketing information, and usage information of new agricultural production technologies [16]. This helps agricultural cooperatives promote new technology. In other words, internal trust enables agricultural cooperatives’ members to quickly accept the green production concept conveyed by the agricultural cooperative and adopt the green production technology promoted by the agricultural cooperative. In terms of internal norms, as an informal system generally observed by internal members, norms help to reduce cooperative transaction costs, restrain abusive behavior of internal members, and motivate farmers to implement the sustainable development strategy following the requirements of agricultural cooperatives. In summary, this study argues that the social capital within agricultural cooperatives, consisting of social networks, internal trust, and internal norms, positively influences its members’ IPM technology adoption behavior.

**Hypothesis** **1.** *The social capital within agricultural cooperatives positively motivates IPM technology adoption behavior by its members.*

### 2.2. The Mediating Role of Agricultural Cooperatives’ Members’ IPM Technology Cognition

Social cognitive theory suggests that the social environment acts directly on individuals’ cognitive levels and behavioral choices. Social capital is a particular constitutive form of the social environment that influences individuals’ cognitive levels and behavioral choices [17]. Social networks, internal trust, and norms constitute the social capital within agricultural cooperatives, which is the key socioeconomic environment for their members and influences their cognitive level. The social network within agricultural cooperatives is an essential channel for members to access agricultural production news, marketing information, and knowledge about agricultural technologies. Frequent communication between agricultural members will bring prosperous marketing and agricultural technical information to them, affecting members’ cognitive level on the instructions and effectiveness of relevant green production technologies. The improvement of trust level among agricultural cooperative members will further promote their communication and enhance the accuracy and acceptability of technical information, which will also act on the cognitive level of members [10]. Internal norms can influence their cognition by encouraging and rewarding the green production behaviors and punishing the non-green production behaviors of the members. The adoption of new agricultural technologies is ultimately promoted.

In addition, previous studies also found that farmers’ cognition of new technologies is a prerequisite for their adoption and plays a vital role in the adoption of new technologies [18,19]. It has been pointed out that the lower the level of farmers’ cognition of the value of utilization of agricultural waste resources, the lower their willingness to recycle agricultural waste [16]; improving the level of farmers’ cognition of conservation tillage technology will positively affect the adoption of conservation tillage technology [5,20]. Therefore, this study supposes that there is a positive relationship between the level of agricultural cooperative members’ IPM technology cognitive level and IPM technology adoption behavior.

Combining the ideas of previous studies with the research theme of this study, Hypothesis 2 is proposed: Agricultural cooperative members’ IPM technology cognition has a mediating effect between the social capital within agricultural cooperatives and its members’ IPM technology adoption behavior.

## 3. Research Methods

### 3.1. Model Specifications

#### 3.1.1. Benchmark Model

The IPM technology adoption by agricultural cooperative members analyzed in this study is a binary dummy variable. Therefore, following the research by Liu et al. [21], the impact of the social capital within agricultural cooperatives on its members’ IPM technology adoption behavior is analyzed mainly by building a binary Probit model, and the equation is as follows.

(1) $$P\left(Y_{i}=1|Z_{i}\right)=\Phi \left(\tau X_{i}+Z_{i}\beta +\mu_{i}>0|Z_{i}\right)$$

where $Y_{i}$ is a binary dummy variable indicating whether member i has adopted IPM technology, such that a value of 1 means that the member has adopted IPM technology, and 0 otherwise; $X_{i}$ indicates the valuation of the social capital within agricultural cooperatives owned by the agricultural cooperative member i; and $Z_{i}$ represents other control variables affecting the adoption of IPM technology by agricultural cooperative members, including individual characteristics of members (e.g., gender, age, education level), household characteristics (e.g., land area, number of off-farm labor, annual household income), agricultural cooperative characteristics (e.g., time to create, demonstration level, existing capital), and regional variables. τ and β are parameters to be estimated, and $\mu_{i}$ is a random error term.

Considering that adopting IPM technology by agricultural cooperatives’ members is a complex decision-making process, there may be endogeneity problems caused by omitted variables in the process of model setting. Meanwhile, the endogeneity problem may also be caused by potential bidirectional causality between the social capital within agricultural cooperatives and IPM technology adoption by agricultural cooperative members. To be specific, members who adopt IPM technology may have rich social capital themselves, and the participation of such members will significantly improve the social capital within agricultural cooperatives. Therefore, this study introduces instrumental variables based on the Probit model and constructs an IV-probit model to estimate the relationship between the social capital within agricultural cooperatives and IPM technology adoption behavior by its members.

The instrumental variable must be a variable that is correlated with the social capital within agricultural cooperatives but not with the adoption or non-adoption of IPM technology by the members. In this study, we selected the agricultural cooperative scale as the instrumental variable, which indicates the number of agricultural cooperative members in the agricultural cooperative. Moreover, the agricultural cooperative scale variable in this study satisfies the requirement of correlation and homogeneity. Specifically, on the one hand, the expansion of the agricultural cooperative scale within a reasonable range will enhance the social capital within agricultural cooperatives and promote the information exchange and resource access of their members [22]. On the other hand, some studies have also pointed out that too large a scale is not conducive to agricultural cooperatives’ development, and an excessive number of members may not be conducive to building internal trust [23]. Meanwhile, the agricultural cooperative scale does not directly affect members’ adoption of IPM technology. Moreover, the estimation results show that the relationship between the agricultural cooperative scale and social capital within agricultural cooperatives is statistically significant at the 1% level and passes the over-identification test. It indicates that the instrumental variable of “the agricultural cooperative scale” is reasonable.

#### 3.1.2. Mediation Effect Model

The second purpose of this study is to analyze the mechanism by which social capital within agricultural cooperatives influences members’ IPM technology adoption behavior. Based on the previous discussion, this study supposes that the agricultural cooperative members’ IPM technology cognition mediates between the social capital within agricultural cooperatives and its members’ IPM technology adoption behavior. Thus, based on the IV-probit model regression, this study further explores the mechanism by which the social capital within agricultural cooperatives influences members’ IPM technology adoption behavior. Following the study by Wen and Ye [24], the mediating effect of agricultural cooperatives’ members’ IPM technology cognition is estimated by drawing on the stepwise regression method proposed by Baron and Kenny with the following equations [25].

(2) $$Y=cX+e_{1}$$

(3) $$M=aX+e_{2}$$

(4) $$Y=c^{\prime }X+bM+e_{3}$$

where M is the mediating variable, representing the level of cooperative members’ cognition of IPM technology, quantified using a five-level Richter scale. The mediation effect testing procedure is divided into four steps. First, if c is statistically significant, the explanatory variable X directly affects Y. The test continues; otherwise, the analysis stops. Second, if both the coefficients of a and b are significant, the test continues. If one of them is insignificant, it goes directly to the fourth step. Third, if the coefficient $c^{\prime }$ is not significant, it means that M plays a fully mediating role; if the coefficient $c^{\prime }$ is significant and $c^{\prime }<c$, M plays a partially mediating role. Fourth, the Sobel test is conducted according to the results of the second step. If there is a mediating effect, the value is ab, and the proportion of the mediating effect of X through M on Y to the total effect of X on Y is ab/c.

### 3.2. Data

As one of the world’s four most cultivated fruits, citrus has a wide planting area. Its green production behavior broadly impacts human health and the environment, especially in mountainous and hilly regions [21]. According to the National Bureau of Statistics of China [26], in 2020, China’s citrus planting area reached 2.7 million hectares, and its output was 51,219,000 tons, ranking first in the world in both aspects. At the same time, based on the statistical data from NBSC, the total area of citrus in Sichuan, mainly mountainous and hilly, was about 313,300 hectares, with a production of 4.8896 million tons, ranking fourth in China. As a central agricultural province, Sichuan is located in the upper reaches of the Yangtze River, and the green innovation of its agricultural producers affects not only the ecological environment and quality of agricultural products in the region but also the water quality issues in the middle and lower reaches of the Yangtze River.

Therefore, the data used in this study were derived from a field survey conducted between June and October 2020 in the Sichuan province. A multistage sampling procedure was used to select regions, cities, counties, agricultural cooperatives, and members for this survey. First, the Chengdu Plain Economic Zone, Sichuan South Economic Zone, and Sichuan Northeast Economic Zone were selected as the research regions according to the economic development level and citrus production distribution. Second, one to three cities were purposely selected based on the development level of the citrus plantation industry, and one to three counties were selected from each city based on the number of citrus planting cooperatives in the three economic zones. Third, two to six citrus planting cooperatives were randomly selected in each county. Finally, five to eight members were randomly selected in each cooperative. In total, we interviewed 465 farm households from 56 agricultural cooperatives.

### 3.3. Variable Selection and Descriptive Statistics

(1) Independent variables. The independent variable in this study is the social capital within agricultural cooperatives, measured by the social network, internal trust, and internal norms, the connotations of which are described in the previous section, and the specific measurement indicators are shown in Table 1. The indicators were assigned in the order of “strongly disagree”, “disagree”, “average”, “agree”, and “strongly agree”, respectively, using a scale of 1–5. The higher the score, the closer the social network, the higher the level of internal trust, and the stronger the sense of internal normative identity. The mean value of social capital within agricultural cooperatives of the sample members was calculated to be 3.74.
(2) Dependent variables. The dependent variable of this study is the adoption of IPM technologies by the cooperative members, which was measured by a dummy variable regarding existing studies [3]. The dependent variable was measured by asking, “Did you adopt IPM technologies in your agricultural production in 2019” in the questionnaire. The mean value of adoption was 0.55, based on Table 2, which means that more than half of the sample members have adopted IPM technologies in their production.

(3) Control variables. Based on previous studies, this paper selected control variables, including respondents’ individual, household, and cooperative characteristics. Specifically, individual characteristics, such as gender [6], age [3], and education level [5], household characteristics, such as family farming area [4], number of off-farm laborers [27], and annual household income [28], and critical characteristics of cooperatives, such as the establishment time [29], demonstration level (according to the act ‘Opinions on carrying out the construction action of the farmer’s professional cooperative demonstration’ published by Ministry of Agriculture in 2009, the selection principles of demonstration agricultural cooperatives include management democracy, economic strength, the number of members, and social reputation. From low to high, there are four levels of demonstration agricultural cooperatives, including county-level demonstration, city-level demonstration, province-level demonstration, and national-level demonstration), and available capital [30,31] were included. The descriptive and statistical characteristics of the variables in the model are shown in Table 2.

## 4. Empirical Analysis

### 4.1. Analysis of the Influence of Social Capital within Agricultural Cooperatives on Members’ IPM Technology Adoption Behavior

In Table 3, the coefficients of social capital within agricultural cooperatives are statistically significant and positive. Specifically, the coefficient of social capital within agricultural cooperatives in the benchmark model (Probit model) indicates that members’ social capital within agricultural cooperatives significantly promotes the adoption of IPM technology by the members at the 1% level. After considering the potential endogenous issue by adding the instrumental variable (IV-probit model), the effect of social capital within agricultural cooperatives on members’ adoption of IPM technology is still positively significant, but the coefficient becomes larger. This indicates that the use of the Probit model may underestimate the effect of social capital within agricultural cooperatives on members’ adoption of IPM technology when endogeneity is not taken into account. This further illustrates the need to consider the endogeneity issue. Therefore, the interpretation of the model results in this study is based on the estimated results of the IV-probit model.

First, social capital within agricultural cooperatives can positively and significantly influence members’ IPM technology adoption behavior, which validates Hypothesis 1 of this paper. Additionally, the finding is consistent with the results of previous studies [17]. The potential reasons are that rich social capital within agricultural cooperatives accompanied by complex social networks can help members access more interpersonal and information resources. Such resources promote the sharing of expertise and adoption experience of IPM technologies, advance members into the understanding and interest stage of IPM technology diffusion, and provide a good demonstration for eventual adoption. Meanwhile, members with rich social capital within agricultural cooperatives have higher trust in other members, the board chairperson, and the cooperative rules and regulations. Furthermore, members with rich internal social capital have closer cooperative relationships with other members. They are more likely to participate in green production as a collective action, which to some extent will also promote members’ adoption of IPM technologies. In addition, internal norms are also a critical part of social capital within agricultural cooperatives. High-level internal norms create an excellent demonstration effect and constrain members to produce according to the requirements. Thus, internal norms urge members to make behavioral choices consistent with green production, which promotes members’ adoption of green production technologies such as IPM.

In addition, several city dummy variables, namely Meishan, Ziyang, and Yibin, significantly influence the adoption of IPM technology among the cooperative members, indicating that cooperative members in Meishan, Ziyang, and Yibin are more likely to adopt IPM technology compared to members in the other sample cities.

### 4.2. Robustness Testing

We employed a simulated sampling method of the original data, i.e., the bootstrap method, to check the robustness of our results. First, 80% of the subsamples were randomly selected from the total sample. Second, the IV-probit model was used to regress the extracted subsamples. The above steps were repeated 1000 times to obtain 1000 corresponding estimates of coefficients of social capital within agricultural cooperatives. The risk of estimation bias due to omitted variables was assessed by determining the position of the coefficient estimates of social capital within agricultural cooperatives in the distribution map of the coefficient estimates of the subsample. The results show that the coefficient estimates of social capital within agricultural cooperatives regressions obtained using the bootstrap method are concentrated around the total sample coefficient values obtained using the IV-probit model. This indicates that the results obtained by using the IV-probit model are robust.

### 4.3. Mediating Effect Test

Following the steps of the test for mediating effects proposed by Baron and Kenny [25], the possible mediating effects were tested by combining the stepwise regression method. As shown in Table 4, social capital within agricultural cooperatives significantly and positively affects members’ cognition of IPM technology at the 1% level with a coefficient of 0.449. This is consistent with the findings of Su et al. [17]. They pointed out that farmers with rich social capital could acquire knowledge more conveniently and had a more comprehensive cognition of the values that arable land possesses, such as economic production, social security, and ecological conservation. According to the above analysis, members’ cognition of IPM technology positively and significantly influences their adoption of IPM technology at the 1% level. Meanwhile, social capital within agricultural cooperatives positively and significantly influences members’ adoption of IPM technology at the 5% level with a coefficient of 0.074 (less than the total effect, i.e., 0.449). Thus, it can be concluded that members’ cognition of IPM technology significantly affects the relationship between social capital within agricultural cooperatives and members’ adoption of IPM technology.

The empirical results of this study suggest that social capital within agricultural cooperatives positively influences members’ cognition of IPM technologies and thus motivates them to adopt IPM technologies. The possible reason is that when members have abundant internal social capital within cooperatives, members communicate frequently and trust each other [18]. Then, information about IPM technologies spreads rapidly among members. As a result, members’ cognition of the technology is deepened, and their understanding of the technology is improved. Therefore, members are more likely to adopt IPM technologies.

## 5. Conclusions and Policy Implications

Based on social capital theory and social cognition theory, this paper empirically estimated the effect of social capital within agricultural cooperatives on members’ IPM technology adoption behavior from the microscopic perspective of members, taking members of the Sichuan Citrus Planting Cooperative as an example. We also introduced the cognitive variables of IPM technology to reveal the underlying mechanism of social capital within agricultural cooperatives to influence members’ adoption of IPM technology. The results showed that the social capital within agricultural cooperatives positively influenced members‘ adoption of IPM technology, and the level of members’ IPM technology cognition played a partial mediating effect in the process of social capital within agricultural cooperatives influencing members’ IPM technology adoption behavior, accounting for 51.37% of the total effect. The above findings implied that among all optional measures to promote IPM technology diffusion in cooperatives, multi-dimensional accumulation of internal social capital and enhancement of members’ IPM technology cognitive level is a path worthy of attention.

Based on the above analysis results, this paper proposes the following insights to provide guiding suggestions for enriching the social capital within agricultural cooperatives and giving full play to their ability to drive smallholder farmers to adopt green production technologies and realize green production transformation.

First, given that the social capital within agricultural cooperatives positively influenced members‘ adoption of IPM technology, agricultural cooperatives or the government can incentivize agricultural producers with rich social capital to join cooperatives to enrich the social capital of other cooperative members. Specifically, local governments use project support, reputation building, and financial support to purposefully incentivize individuals or organizations with abundant social capital who intend to engage in agricultural production to establish or join cooperatives. Through the exchange of production within cooperatives, the social capital of cooperative members will be increased. Second, given that the cognition of members’ IPM technology played a mediating effect, agricultural cooperatives are encouraged to improve members’ trust in other members and cooperatives as an organization. By actively promoting openness of issues, financial transparency, fair distribution, and conducting extensive internal communication activities, each member can understand and trust the cooperative and other members, providing a mass base for the cooperative to promote IPM technology. Furthermore, agricultural cooperatives are encouraged to give full play to the active role of cooperatives as technology promotion carriers and enhance the level of cognition of new technologies among members. Specifically, strengthening the construction of cooperatives’ internal technical information sharing platform can promote information exchange and experience sharing among members. Furthermore, establishing and implementing a technical training system can strengthen training and technical guidance for members on IPM technology and other related knowledge.

## Figures and Tables

**Table 1:** Evaluation index of social capital within agricultural cooperatives.

Dimensionality	Metrics	Mutator Methods
Social networks	Get along well with other members	The specific indicators were assigned weights according to the values corresponding to the options of the respondent’s answers to the questions and summed according to the entropy method. The higher the final score, the richer social capital within agricultural cooperatives.
	Communicate frequently with other members	
	High frequency of participation in collective activities of the cooperative	
Internal trust	High level of trust in other members	
	High level of trust in the president of the cooperative	
	High level of trust in the rules and regulations of the cooperative	
Internal specifications	Consideration of other members’ opinions in production and management	
	Consider the interests of other members and the cooperative as a whole in production and management	
	Proactive in making suggestions for other members in the production and management process	

Note: Measurements are also specific questions in questionnaires, measured by consulting respondents on the extent to which they agree with the above statement, with options including 1—strongly disagree, 2—disagree, 3—general, 4—agree, and 5—strongly agree.

**Table 2:** Variable selection and description statistics.

Variables		Variable Definition	Mean	S.D.
Independent variable	IPM adoption	1 if a farmer adopted IPM in 2019, 0 otherwise	0.55	0.50
Dependent variable	The social capital within agricultural cooperatives	Level of the social capital within agricultural cooperatives based on Table 1 (scores)	3.74	0.66
Control variables				
Individual characteristics	Gender	1 if the household head is male, 0 otherwise	0.71	0.45
	Age	Age of the household head (years)	55.25	10.09
	Education	Formal education of the household head (years)	7.56	3.63
Households’ characteristics	Farm size	Total size of citrus-planting orchards (mu)	6.06	4.93
	Non-farm workers	Number of household members who are non-farm workers	4.22	1.71
	Household income	Annual total income of the farmer’s household in 2019 (1000 yuan)	309.90	974.60
Cooperatives’ characteristics	Time of creation	Duration of cooperative establishment as of the end of 2019 (years)	6.40	3.42
	Demonstration level	Non-model = 1, county-level model = 2, municipal-level model = 3, provincial-level model = 4, national-level model = 5	2.25	1.33
	Existing capital	The current total capital of cooperatives in 2019 (million yuan)	521.35	1117.30
Instrumental variable	Cooperative scale	Number of cooperative members at the end of 2019	167.35	203.58
Mediation variable	Cognitive level of IPM	Do not know = 1, know a little = 2, average = 3, know better = 4, know very well = 5	4.09	0.64
Region variable	Region_Chendu	1 if a farmer is located in Chengdu, 0 otherwise	0.10	0.30
	Region_Meishan	1 if a farmer is located in Meishan, 0 otherwise	0.32	0.47
	Region_Nanchong	1 if a farmer is located in Nanchong, 0 otherwise	0.11	0.31
	Region Ziyang	1 if a farmer is located in Ziyang, 0 otherwise	0.20	0.40
	Region_Neijiang	1 if a farmer is located in Neijiang, 0 otherwise	0.17	0.37
	Region_Yibin	1 if a farmer is located in Yibin, 0 otherwise	0.11	0.31

Note: 1 mu =1/15 hectare.

**Table 3:** The estimation results of Probit model and IV-probit model.

Variables	Probit		IV-Probit	
	Coefficient	Standard Error	Coefficient	Standard Error
Social capital within agricultural cooperatives	0.460 ***	0.123	1.874 ***	0.283
Gender	0.011	0.151	0.199	0.137
Age	−0.016 **	0.008	0.010	0.014
Education	0.029	0.022	0.030	0.031
Land area	−0.011	0.016	−0.006	0.013
Off-farm labor	0.011	0.040	−0.004	0.033
Household income	0.001	0.001	0.000	0.001
Time of creation	−0.033	0.022	0.015	0.031
Demonstration level	0.059	0.053	−0.026	0.063
Existing capital	0.001 **	0.000	0.001	0.000
Region_Meishan	0.198	0.246	0.455 **	0.196
Region_Nanchong	−0.520	0.328	0.803	0.621
Region_Ziyang	−0.427	0.279	1.046 *	0.628
Region_Neijiang	−0.566 **	0.276	0.507	0.556
Region_Yibin	−0.307	0.300	0.941 *	0.538
Constant	−0.790	0.785	−7.726	1.914
Wald	—		533.71	
R^2^	0.195		—	

Note: *, **, and *** represent significant at the level of 1%, 5%, and 10%, respectively. The values in parentheses are standard errors.

**Table 4:** The results of mediating effect estimation.

Variables	Effect Decomposition		Total Effect
	Cognitive Level of IPM	Adoption of IPM	Adoption of IPM
Social capital within agricultural cooperatives	0.449 (0.049) ***	0.074 (0.041) **	0.153 (0.039) ***
Cognitive level of IPM	—	0.175 (0.036) ***	—
Control variables	Controlled	Controlled	Controlled
R^2^	0.2505	0.2693	0.2315
Adjusted R^2^	0.2255	0.2432	0.2058
F-value	10.01 ***	10.32 ***	9.02 ***

Note: ** and *** represent significance at the level of 5% and 1%, respectively. The values in parentheses are standard errors.

## Data Availability

The authors may provide raw data if necessary.
